# The cellular and circuit basis for evolutionary change in sensory perception in mormyrid fishes

**DOI:** 10.1038/s41598-017-03951-y

**Published:** 2017-06-19

**Authors:** Alejandro Vélez, Tsunehiko Kohashi, Anan Lu, Bruce A. Carlson

**Affiliations:** 1 Department of Biology, Washington University in St. Louis, St. Louis, MO USA; 2 Division of Biological Science, Graduate School of Science, Nagoya University, Nagoya, Japan

## Abstract

Species differences in perception have been linked to divergence in gross neuroanatomical features of sensory pathways. The anatomical and physiological basis of evolutionary change in sensory processing at cellular and circuit levels, however, is poorly understood. Here, we show how specific changes to a sensory microcircuit are associated with the evolution of a novel perceptual ability. In mormyrid fishes, the ability to detect variation in electric communication signals is correlated with an enlargement of the midbrain exterolateral nucleus (EL), and a differentiation into separate anterior (ELa) and posterior (ELp) regions. We show that the same cell types and connectivity are found in both EL and ELa/ELp. The evolution of ELa/ELp, and the concomitant ability to detect signal variation, is associated with a lengthening of incoming hindbrain axons to form delay lines, allowing for fine temporal analysis of signals. The enlargement of this brain region is also likely due to an overall increase in cell numbers, which would allow for processing of a wider range of timing information.

## Introduction

The evolution of behavior and perception is related to evolutionary changes in the brain. Evolutionary differences in sensory perception have been described in many clades of animals and, in some cases, correlated to differences in gross brain anatomy^1–10^. The anatomical and physiological basis of evolutionary change in sensory processing at cellular and circuit levels, however, has received less attention.

**Figure 1:**
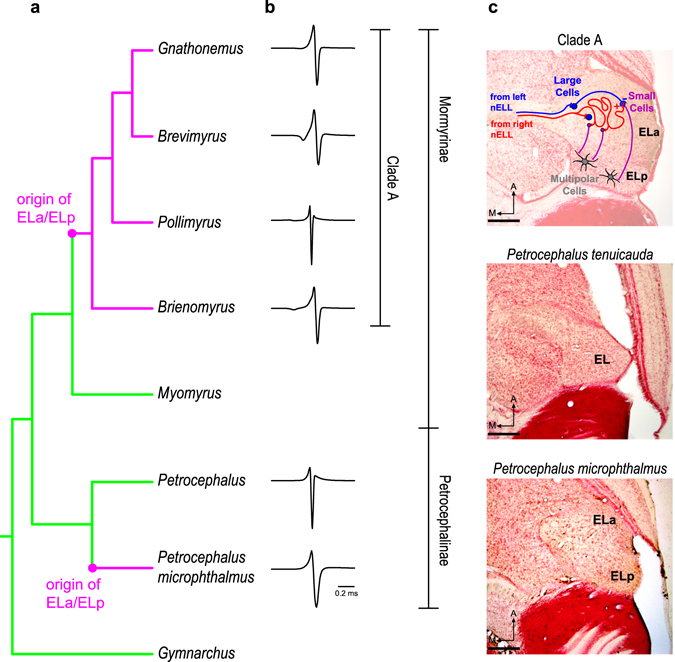
The perceptual ability to detect variations in EOD waveform is associated with parallel evolutionary changes of the central electrosensory system in mormyrids. Based on a parsimonious reconstruction of extant taxa^12^, the most likely ancestral state of the exterolateral nucleus of the midbrain (EL) is small and undifferentiated (green) (**a**,**c**). In clade A and *P*. *microphthalmus*, however, this nucleus is enlarged and subdivided into anterior (ELa) and posterior (ELp) regions (magenta) (**a**,**c**). Only species with ELa/ELp are sensitive to EOD waveform variation (i.e., the shape of the electric pulses used in communication) (**b**). In clade A, the first stages of EOD waveform analysis are performed in ELa (**c**). Cells from the hindbrain nucleus of the electrosensory lateral line lobe (nELL) project bilaterally to ELa (red and blue). Upon entering ELa, the nELL axons synapse onto large inhibitory cells (blue) and then follow a long and tortuous path, synapsing onto numerous small cells (purple) throughout their length. Large inhibitory cells project onto small cells, establishing a delay-line anti-coincidence detection mechanism by which small cells analyze EOD waveform. Small cells then project to multipolar cells (gray) in ELp that are sensitive to inter-pulse intervals (IPIs). Cladogram in (**a**) is based on consensus trees^43, 44^ and includes genera of the species used in this study, as well as the genera *Myomyrus* and *Gymnarchus*, which were important for previous reconstructions of the EL-ELa/ELp trait^12^. EOD waveforms in (**b**) are from species used in the present study (from top to bottom): *Gnathonemus petersii*, *Brevimyrus niger*, *Pollimyrus adspersus*, *Brienomyrus brachyistius*, *Petrocephalus tenuicauda*, and *Petrocephalus microphthalmus*. Photomicrographs in (**c**) are from 50-µm horizontal sections of the brain at the level of the midbrain and scale bars represent 200 µm. A: anterior. M: medial.

We addressed this question in weakly electric fishes of the family Mormyridae. Mormyrids communicate using pulse-type electric organ discharges (EODs; Fig. 1). EOD waveform (i.e., the shape of the electric pulse in a plot of voltage vs. time) is species-specific, highly stereotyped, and provides information about sender identity^11^. Species from two mormyrid lineages can detect subtle variations in EOD waveform^12^. One of these lineages is represented by only one known extant species in the subfamily Petrocephalinae: *Petrocephalus microphthalmus* (Fig. 1). The other lineage belongs to the subfamily Mormyrinae, is known as “clade A”, and includes over 175 species^12, 13^. Interestingly, the perceptual ability to detect EOD waveform variation likely evolved independently in both lineages and is associated with changes in peripheral receptor anatomy and physiology, and in the gross anatomy of the central electrosensory system^12, 14, 15^ (Fig. 1). Here, we refer to this perceptual ability and to the associated changes in the electrosensory system as the derived states of these traits, as this is the most likely evolutionary scenario given parsimonious reconstructions based on data from extant taxa^12^.

Knollenorgans, electroreceptors on the surface of the skin that detect electric communication signals, are distributed throughout the surface of the body in species sensitive to EOD waveform variation^12, 14^. In these species, knollenorgans respond to electrosensory stimulation with a single time-locked spike^14, 16^. In species unable to detect EOD waveform variation, knollenorgans are clustered in three rosettes on both sides of the head^13, 14^. These electroreceptors do not fire spikes but produce spontaneously oscillating potentials. When stimulated, oscillating receptors respond with an increase in oscillation amplitude and with a phase reset that results in transient synchrony across receptors^14^.

In the central electrosensory system, the most likely ancestral state of the midbrain exterolateral nucleus (EL) is small and undifferentiated^12^ (Fig. 1). In contrast, EL is enlarged and subdivided into separate anterior (ELa) and posterior (ELp) regions in clade A and *P*. *microphthalmus* ^12^ (Fig. 1). Importantly, both EL and ELa/ELp are devoted to processing communication signals, as they only respond to the EODs of neighboring fish, not to the fish’s own EOD^17–21^. Studies of clade-A species have provided valuable insight into the processing of electric communication signals in ELa/ELp^22^ (Fig. 1). Knollenorgans project ipsilaterally to the nucleus of the electrosensory lateral line lobe (nELL) in the hindbrain^18, 23–27^. Cells from nELL project to ELa through the lateral lemniscus; most projections are contralateral though some are ipsilateral or bilateral^18, 26, 28^. These projections synapse onto two cell types in ELa: small cells and large cells. Small cells perform the first stage of EOD waveform analysis based on a delay-line anti-coincidence detection mechanism^28, 29^. The circuit includes inhibition from large cells onto small cells and excitation from nELL cells onto small cells via axons that follow long and convoluted paths. These axons synapse onto multiple small cells along their length, thus establishing delay lines that determine the relative timing of inhibition and excitation onto small cells, generating submillisecond sensitivity to variation in EOD waveform. Small cells in ELa project to multipolar cells in ELp that are sensitive to the inter-pulse intervals (IPIs) between EODs^30^. IPIs are variable and depend on social context^31, 32^.

While IPI sensitivity is common across mormyrids, only species with ELa/ELp are sensitive to EOD waveform variation^12, 14^. However, the microcircuitry of this midbrain region has only been studied in clade-A species. To understand the underlying physiological and anatomical mechanisms of evolutionary change in sensory perception, we investigated how the neural circuitry in ELa/ELp of *P*. *microphthalmus* and in the small and undifferentiated EL of *Petrocephalus tenuicauda* relate to the neural circuitry in ELa/ELp of clade A. We used neuronal tract tracing, immunohistochemistry, and electrophysiology to compare the anatomy and physiology of the unknown EL and ELa/ELp neural circuits of *Petrocephalus* spp. to the known ELa/ELp neural circuit of clade-A species. Specifically, we investigated the neuronal projections from the hindbrain to these midbrain circuits, the types of cells found in these neural circuits and their morphology, the synaptic connections among those cells, and the processing of electrosensory information within these circuits.

## Results

### Contralateral bias in projections from hindbrain cells to ELa and to the anterior end of EL

**Figure 2:**
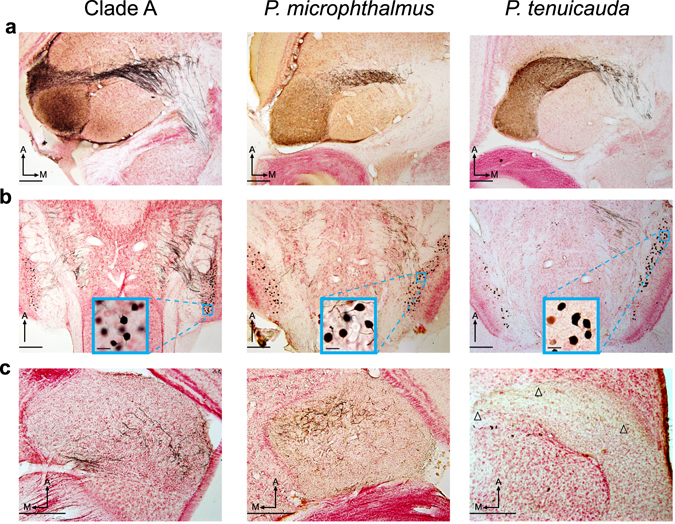
Neurons from the hindbrain nELL project bilaterally to the midbrain in all three lineages. Iontophoretic injections of neuronal tract tracers in the left ELa of a clade-A species (*B*. *niger*, left) and *P*. *microphthalmus* (center), and in the anterior end of the left EL in *P*. *tenuicuda* (right) labeled axonal projections through the lateral lemniscus (**a**) and backfilled somas in the left and right hindbrain nELL (**b**). The majority of backfilled neurons were in the right nELL in the three lineages (Clade A: *B*. *brachyistius* n = 61stained cells total, 60% contralateral; *B*. *niger* subject 1: n = 494, 67% contralateral; *B*. *niger* subject 2: n = 559, 69% contralateral; *B*. *niger* subject 3: n = 308, 64% contralateral; *G*. *petersii*: n = 204, 67% contralateral. *P*. *microphthalmus* subject 1: n = 156 stained cells total, 59% contralateral; *P*. *microphthalmus* subject 2: n = 481, 68% contralateral. *P*. *tenuicauda* subject 1: n = 10 stained cells total, 70% contralateral; *P*. *tenuicauda* subject 2: n = 523, 71% contralateral; *P*. *tenuicauda* subject 3: n = 21, 75% contralateral). (**c**) Stained axonal projections in the right ELa and anterior end of the right EL show that some projections from the hindbrain are bilateral. Insets in (**b**) show close-ups of stained somas of nELL neurons contralateral to the injection site. All photomicrographs are from 50-µm horizontal sections of the brain. Scale bars in the insets in (**b**) represent 20 µm; all other scale bars represent 200 µm. Arrowheads point to labeled axonal projections in the contralateral EL of *P*. *tenuicauda*. All photomicrographs were taken from the same subject in each lineage. The injection sites are not visible in these sections. With respect to the photomicrographs in (**a**), injection sites were: 250 µm dorsal and towards the lateral edge of ELa in clade A; 400 µm dorsal and towards the anterior edge of ELa in *P*. *microphthalmus*; 250 µm dorsal and towards the anterolateral edge of EL in *P*. *tenuicauda*. A: anterior. M: medial.

In clade-A species, the only inputs to ELa are from cells in the ipsi- and contra-lateral nELL of the hindbrain^18, 26, 28^. We asked whether the inputs to EL of *P*. *tenuicauda* and ELa of *P*. *microphthalmus* are similar to those into ELa of clade-A species. Injections of neuronal tract tracers into the left ELa of clade-A species and *P*. *microphthalmus*, and into the anterior end of EL in *P*. *tenuicauda*, retrogradely labeled cells in the ipsilateral and contralateral nELL (Fig. 2a,b). In all cases, we found more labeled cells in the contralateral nELL (Fig. 2b; clade A: n = 3 species, 5 subjects, 60–69% of quantified stained cells found contralaterally; *P*. *microphthalmus*: n = 2 subjects, 59% and 68%; *P*. *tenuicauda*: n = 3 subjects, 70–75%). Consistent with bilateral projections from the hindbrain to the midbrain, we also found labeled axonal projections, but no stained somas, in the right ELa of clade-A species and *P*. *microphthalmus* and in the anterior end of the right EL of *P*. *tenuicauda* (Fig. 2c).

**Figure 3:**
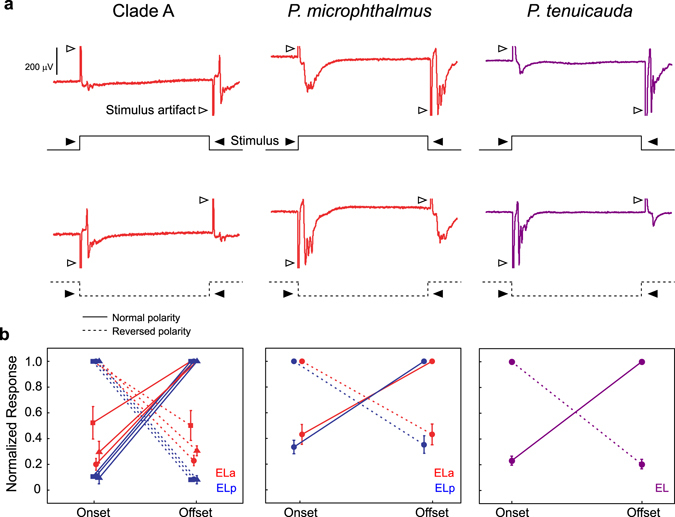
Sensory input to the midbrain is biased towards stimulation of electroreceptors on the contralateral side of the body in all three lineages. (**a**) Representative mean evoked potentials (n = 10 traces) from the left ELa (red traces) of a clade A species (*G*. *petersii*, left) and *P*. *microphthalmus* (center), and from the left EL (purple traces) of *P*. *tenuicauda* (right) in response to long square pulses delivered with normal polarity (top row, stimulus depicted by full lines) and reversed polarity (bottom row, stimulus depicted by dashed lines). In our experimental set-up (see Methods), the leading edge of a square pulse with normal polarity stimulates knollenorgans on the left side of the body, while the trailing edge stimulates knollenorgans on the right side of the body. The opposite is true for square pulses delivered with reversed polarity. Both traces come from the same subject for each lineage. (**b**) We measured the peak-to-peak amplitude of the evoked potential in response to each edge of the stimulus and normalized them to the maximum value for each fish tested and each stimulus polarity (normal: solid lines, reversed: dashed lines); for clade A and *P*. *microphthalmus*, we normalized responses separately for ELa (red symbols) and ELp (blue symbols). The amplitude of the evoked potentials depended on side of stimulation (Clade A: *B*. *brachyistius*: n = 2, F_1,7_ = 1377.6, p < 0.0001; *B*. *niger*: n = 2, F_1, 7_ = 304.1, p < 0.0001; *G*. *petersii*: n = 3, F_1,14_ = 2716.8, p < 0.0001; *P*. *microphthalmus*: n = 3, F_1,14_ = 322.7, p < 0.0001; *P*. *tenuicauda*: n = 7 fish, 10 recordings; F_1,30_ = 1390.3, p < 0.0001), but not on order of stimulation (Clade A: *B*. *brachyistius*: n = 2, F_1,7_ = 0.071, p = 0.80; *B*. *niger*: n = 2, F_1, 7_ = 0.002, p = 0.97; *G*. *petersii*: n = 3, F_1,14_ = 0.002, p = 0.96; *P*. *microphthalmus*: n = 3, F_1,14_ = 0.02, p = 0.88; *P*. *tenuicauda*: n = 7 fish, 10 recordings; F_1,30_ = 0.45, p = 0.51). Each symbol in (**b**) represents the mean normalized amplitude and the error bars represent the SEM. Clade A species tested include *B*. *brachyistius* (squares), *B*. *niger* (triangles), and *G*. *petersii* (circles).

In clade-A species, knollenorgans on each side of the body project ipsilaterally to the nELL^14, 16, 33^. Thus, the greater number of contralateral projections from nELL to ELa result in more sensory information reaching ELa from the contralateral side of the fish’s body^18, 23, 24, 28, 34^. We asked whether this pattern is common across lineages. Importantly, knollenorgans respond to inward current transients; thus, knollenorgans on one side of the body respond to rising edges of an electrosensory stimulus, whereas knollenorgans on the opposite side of the body respond to falling edges^14, 16, 33^. We obtained *in vivo* evoked potentials from the left ELa/ELp in clade-A species and *P*. *microphthalmus*, and from the left EL in *P*. *tenuicauda*, in response to artificially long (50ms) monophasic square pulses, for which the responses to each edge can be easily separated (Fig. 3). Pulses were delivered with normal or reversed polarity. In our experimental setup (see Methods), knollenorgans on opposite sides of the body are stimulated by different edges of normal- and reversed-polarity stimuli. With normal-polarity stimuli, knollenorgans on the left side of the body (ipsilateral) experienced inward current transients with the leading edge of the square pulse, while knollenorgans on the right side of the body (contralateral) did so with the trailing edge. Conversely, square pulses delivered with reversed polarity resulted in stimulation of the contralateral side of the body by the leading edge, and ipsilateral stimulation by the trailing edge. We asked whether the amplitude of the evoked potential in response to each stimulus edge was influenced by the side of body stimulation (contralateral vs. ipsilateral) and by the order of stimulation (leading vs. trailing).

All lineages showed a similar pattern (Fig. 3). Evoked potentials were stronger in response to contralateral stimulation (repeated measures ANOVA clade A: four species, all p < 0.0001; *P*. *microphthalmus*: n = 3, F_1,14_ = 322.7, p < 0.0001; *P*. *tenuicauda*: n = 7 fish, 10 recordings; F_1,30_ = 1390.3, p < 0.0001). However, the amplitude of evoked potentials did not depend on which side of the body was stimulated first (repeated measures ANOVA clade A: four species, all p > 0.07; *P*. *microphthalmus*: n = 3, F_1,14_ = 0.02, p = 0.88; *P*. *tenuicauda*: n = 7 fish, 10 recordings; F_1,30_ = 0.45, p = 0.51). These results are consistent with the contralateral bias in hindbrain projections to the midbrain and reveal that more sensory input from the contralateral side of the body reaches the midbrain in all three lineages.

### Large inhibitory cells project to smaller cells within ELa and the anterior end of EL

**Figure 4:**
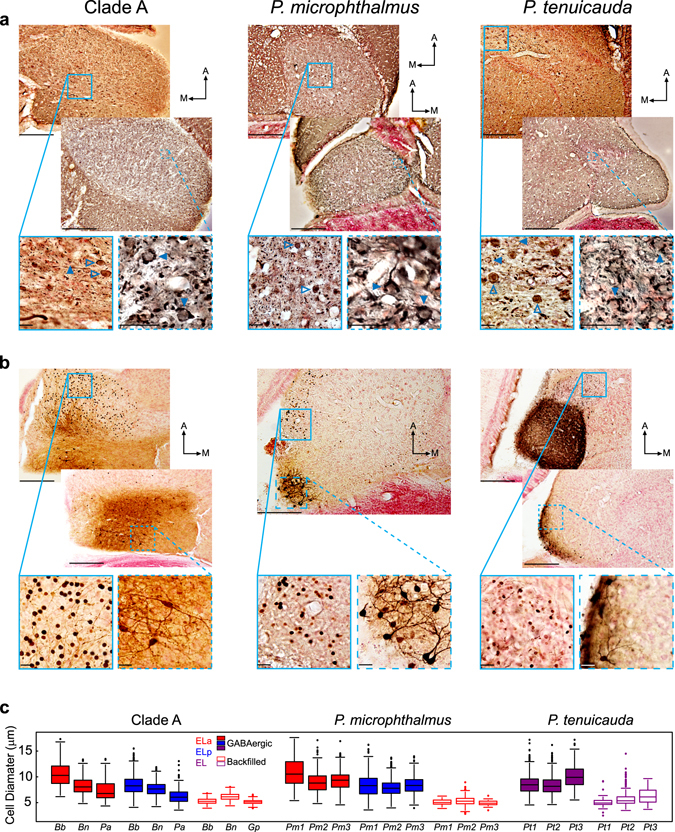
The three same basic types of neurons are found in ELa/ELp of clade-A species and *P*. *microphthalmus*, and in EL of *P*. *tenuicauda*. (**a**) GABA immunohistochemistry revealed inhibitory neurons in ELa and ELp of clade-A species (left) and *P*. *microphthalmus* (center), and throughout EL and the lateral lemniscus of *P*. *tenuicauda* (right). The images on the bottom show enlarged views taken from the boxes and show stained somas (open arrowheads) and terminals onto unstained cell bodies (filled arrowheads). (**b**) Iontophoretic injections in ELp of clade-A species and *P*. *microphthalmus*, and in the posterior end of EL of *P*. *tenuicauda* reveal multipolar cells in ELp and the posterior end of EL, and backfilled adendritic small cells in ELa and the anterior end of EL. Images on the bottom are enlarged views taken from the boxes and show stained somas of backfilled small cells (left), and multipolar cells (right). All photomicrographs in (**a**) and (**b**) are from 50-µm horizontal sections through the midbrain. (**c**) Cell diameters of GABAergic cells (filled boxes) and backfilled cells (open boxes) after injections of neuronal tract tracers in ELp and the posterior end of EL. Cell diameters in (**c**) were measured in ELa (red) and ELp (blue) of clade-A species and *P*. *microphthalmus*, and in EL (purple) of *P*. *tenuicauda*. Data of GABAergic cells were obtained from one *B*. *brachyistius* (*Bb*), one *B*. *niger* (*Bn*), and one *P*. *adspersus* (*Pa*) for clade A, from three individuals of *P*. *microphthalmus* (*Pm1*-*3*), and from three individuals of *P*. *tenuicauda* (*Pt1*-*3*). Data of backfilled cells that project to multipolar cells were obtained from one *B*. *brachyistius* (*Bb*), one *B*. *niger* (*Bn*), and one *G*. *petersii* (*Gp*) for clade A, from three individuals of *P*. *microphthalmus* (*Pm1*-*3*), and from three individuals of *P*. *tenuicauda* (*Pt1*-*3*). In (**a**) and (**b**), scale bars in top photomicrographs represent 200 µm and scale bars in the enlarged photomicrographs represent 20 µm. Clade-A species represented are *B*. *brachyistius* (left) and *B*. *niger* (right) in (**a**) and *B*. *brachyistius* in (**b**). Photomicrographs in (**a**) come from one *B*. *brachyistius* (left) and one *B*. *niger* (right) for clade A, one *P*. *microphthalmus*, and two *P*. *tenuicauda*. Photomicrographs in (**b**) come from one *B*. *niger*, one *P*. *microphthalmus*, and two subjects of *P*. *tenuicauda*. The injection sites are not visible in these sections. Injection sites in (**b**) were: towards the middle of ELp and 350 µm ventral with respect to the top photomicrograph from clade A; towards the lateral and posterior edge of ELp and 350 µm ventral relative to the photomicrograph of *P*. *microphthalmus*; towards the medial and posterior edge of EL and 150 µm ventral with respect to the top photomicrograph of *P*. *tenuicauda*; and towards the lateral and posterior edge of EL and 150 µm ventral relative to the bottom photomicrograph of *P*. *tenuicauda*. A: anterior. M: medial.

EOD waveform analysis in clade-A species is mediated by an anti-coincidence detection mechanism in a circuit that includes direct inhibition and delayed excitation^28, 29^. We used GABA immunhistochemistry to identify GABAergic neurons and their synaptic targets in the EL and ELa/ELp neural circuits (Fig. 4a). GABAergic cells were observed in ELa and ELp of clade-A species and *P*. *microphthalmus*. The diameters of inhibitory somas in ELa of both lineages were similar (Non-outlier ranges of stained cell diameters in µm: *Brienomyrus brachyistius* = 6.16–16.77, n = 165; *Brevimyrus niger* = 4.81–12.61, n = 238; *Pollimyrus adspersus* = 4.34–12.59, n = 188; *P*. *microphthalmus* subject 1 = 5.37–17.55, n = 84; *P*. *microphthalmus* subject 2 = 4.79–13.67, n = 305; *P*. *microphthalmus* subject 3 = 4.53–13.78, n = 305) and consistent with previous reports of inhibitory large cells in ELa of clade-A species^28, 35^ (Fig. 4a,c). We found calyx-like GABAergic terminals onto smaller, unstained cells in ELa of both lineages (Fig. 4a). Inhibitory somas in ELp generally had smaller diameters than those in ELa (Non-outlier ranges of stained cell diameters in µm: *B*. *brachyistius* = 4.10–13.07, n = 1023; *B*. *niger* = 4.22–11.10, n = 686; *P*. *adspersus* = 3.55–9.30, n = 314; *P*. *microphthalmus* subject 1 = 3.56–13.92, n = 404; *P*. *microphthalmus* subject 2 = 3.94–11.68, n = 1345; *P*. *microphthalmus* subject 3 = 4.48–12.59, n = 1303) and were similar in size to the somas of multipolar cells in ELp of clade-A species^27, 35, 36^ (Fig. 4a,c). We found punctate terminals surrounding unstained somas in ELp of both lineages, consistent with inhibitory interactions among multipolar cells in ELp of clade-A species^35^.

In *P*. *tenuicauda*, we found GABAergic cells throughout EL and the lateral lemniscus (Fig. 4a). The range of diameters of these inhibitory somas spanned the range of diameters found throughout ELa and ELp of clade-A species and *P*. *microphthalmus* (Non-outlier ranges of stained cell diameters in µm: *P*. *tenuicauda* subject 1 = 4.49–12.90, n = 761; subject 2 = 4.58–12.58, n = 1925; subject 3 = 5.48–15.33, n = 772). Typically, larger stained cells were found in the lateral lemniscus and towards the anterior end of EL, while smaller cells were found towards the posterior end of EL (Fig. 4a). We found calyx-like GABAergic terminals on the somas of smaller, unstained cells in the anterior end of EL and in the lateral lemniscus, and punctate terminals around unstained somas towards the posterior end of EL (Fig. 4a).

### Small cells in ELa and in the anterior end of EL project to multipolar cells

The only output from ELa of clade-A species is from small cells that synapse onto multipolar cells in ELp^28^. Here, we investigated the projections to ELp in clade-A species and *P*. *microphthalmus* and to the posterior end of EL in *P*. *tenuicauda*. Injections of neuronal tract tracers into ELa of clade-A species and *P*. *microphthalmus* labeled axonal projections into ELp (Fig. 2a). Iontophoretic injections of neuronal tract tracers into ELp of both lineages labeled multipolar cells in ELp and retrogradely labeled cells in ELa (Fig. 4b). Backfilled cells in ELa of both lineages were adendritic and their somas had similar diameters (Non-outlier ranges of stained cell diameters in µm: *B*. *brachyistius* = 3.92–6.77, n = 130; *B*. *niger* = 4.4–8.01, n = 561; *G*. *peterii* = 4.09–6.18, n = 532; *P*. *microphthalmus* subject 1 = 4.07–5.68, n = 41; *P*. *microphthalmus* subject 2 = 3.23–7.32, n = 1303; *P*. *microphthalmus* subject 3 = 3.84–6.22, n = 145). These cells were smaller than GABAergic cells in ELa and ELp (Fig. 4c) and similar in size to the small cells previously described in clade-A species^28, 29, 34, 37^.

In *P*. *tenuicauda*, iontophoretic injections of neuronal tract tracers into the anterior end of EL labeled axonal projections into the posterior end of EL (Fig. 2a). Neuronal tract tracers injected into the posterior end of EL revealed multipolar cells throughout EL and adendritic cells toward the anterior end of EL and in the lateral lemniscus (Fig. 4b). The sizes of labeled adendritic cells in *P*. *tenuicauda* (Non-outlier ranges of stained cell diameters in µm: subject 1 = 3.76–6.35, n = 63; subject 2 = 3.43–7.77, n = 156; subject 3 = 4.10–10.05, n = 204) were similar to those found in ELa of clade-A species and *P*. *microphthalmus* and tended to be smaller than the GABAergic cells (Fig. 4c).

### Sensitivity to inter-pulse intervals (IPIs) arises in ELp and towards the posterior end of EL

**Figure 5:**
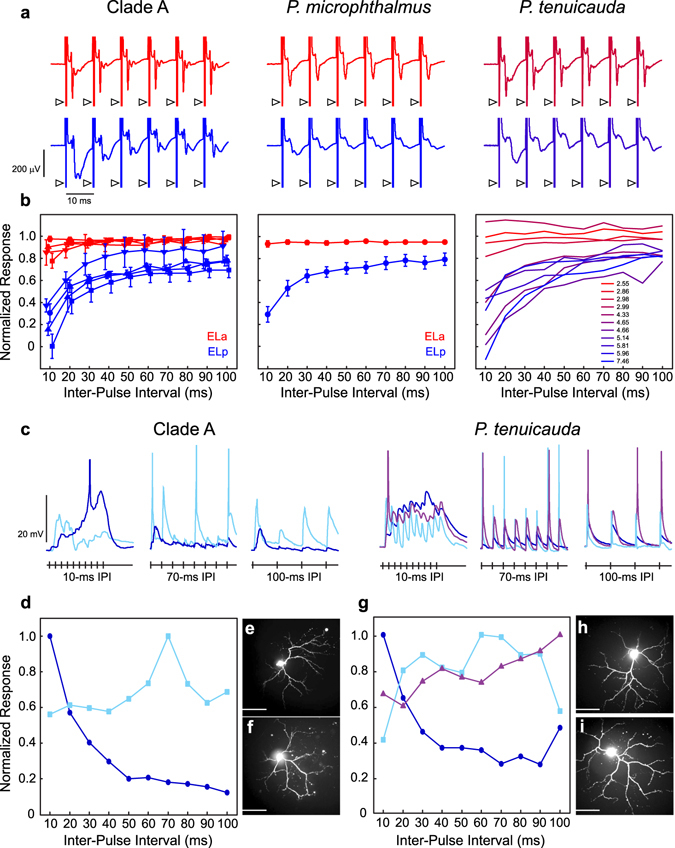
Inter-pulse interval processing arises in ELp of clade-A species and *P*. *microphthalmus*, and in the posterior end of EL in *P*. *tenuicauda*. (**a**) Representative mean evoked potentials (n = 10 traces) from ELa (red) and ELp (blue) of clade A (left) and *P*. *microphthalmus* (center), and towards the anterior (top) and posterior (bottom) ends of EL in *P*. *tenuicauda* (right), obtained *in vivo* in response to electrosensory pulse trains with inter-pulse intervals of 10ms. Arrowheads denote stimulus artefacts. (**b**) We averaged the peak-to-peak amplitude of the evoked potential in response to the second through tenth pulses of the stimulus and normalized it to the peak-to-peak amplitude of the evoked potential in response to the first pulse of the stimulus (see Methods). For clade A (left) and *P*. *microphthalmus* (center), we normalized responses separately for ELa (red symbols) and ELp (blue symbols) and figure symbols represent the mean normalized amplitude and the error bars represent the SEM. The amplitude of evoked potentials attenuates at short IPIs in ELp, but not ELa, of clade-A species (*B*. *brachyistius*: squares, n = 6, F_9,95_ = 4.29, p < 0.001; *B*. *niger*: triangles, n = 4, F_9,57_ = 11.29, p < 0.0001; *P*. *adspersus*: circles, n = 3, F_9,38_ = 11.93, p < 0.0001; *G*. *petersii*: inverted triangles, n = 3, F_9,38_ = 2.00, p = 0.06) and *P*. *microphthalmus* (n = 6, F_9,95_ = 10.80, p < 0.0001). In EL of *P*. *tenuicauda*, evoked potentials attenuate towards the posterior end of EL, but not towards the anterior end. For *P*. *tenuicauda*, the color code represents the latency (in ms) to the first negative peak of the evoked potential in response to a 0.5-ms bipolar square pulse, from the shortest latency in red to the longest in blue. These latencies span those previously reported in ELa and ELp of clade-A species (ELa: 3.7 ms, ELp: 8.2 ms) and *P*. *microphthalmus* (ELa: 2.8 ms, ELp: 7.4 ms)^21^. (**c**) Representative response traces of neurons sensitive to inter-pulse intervals in ELp of *B*. *niger* (left) and the posterior end of EL in *P*. *tenuicauda* (right). *In vitro* whole-cell recordings were obtained in response to electrical stimulation of the lateral lemniscus. Traces depict responses to pulse trains with IPIs of 10ms, 70 ms, and 100 ms. Based on the tuning curves (**d**,**g**, see Methods), neurons were classified as high-pass (dark blue), band-pass (light blue), and low-pass (purple). Neurons sensitive to IPIs in *B*. *niger* (**e**,**f**) and *P*. *tenuicauda* (**h**,**i**) are morphologically similar and highly dendritic, as evidenced by confocal fluorescence imaging (see Methods). Scale bars in photomicrographs represent 50 µm.

In clade-A species, the first stage of IPI analysis takes place in ELp^30^. We asked whether IPI sensitivity also arises in ELp of *P*. *microphthalmus* and EL of *P*. *tenuicauda*. In response to electrosensory stimulation with pulse trains having IPIs between 10 ms and 100 ms, the amplitude of *in vivo* evoked potentials attenuated at short IPIs in ELp, but not ELa of clade-A species (repeated measures ANOVA: three species with p < 0.001, one with p = 0.06) and *P*. *microphthalmus* (repeated measures ANOVA: n = 6, F_9,95_ = 10.80, p < 0.0001) (Fig. 5a,b). These results are consistent with IPI sensitivity arising in ELp^30^.

In EL of *P*. *tenuicauda*, the waveform and latency of *in vivo* evoked potentials vary depending on the location of the recording electrode: evoked potentials are broader and have longer latencies towards the posterior end of EL^21^. The shape and latency of evoked potentials obtained towards the anterior end resemble those of ELa in clade-A species and *P*. *micropthalmus*, while evoked potentials obtained towards the posterior end resemble those of ELp^21^. To avoid any bias in our analyses, here we used the latency of the evoked potential as a proxy for the location of the recording electrode along an anterior-posterior axis of EL. In response to pulse trains with short IPIs, evoked potentials obtained towards the posterior end of EL attenuated whereas those obtained towards the anterior end showed little to no attenuation (repeated measures ANOVA: n = 8 fish, 11 recordings; F_9,83_ = 4.38, p = 0.0001; Fig. 5a,b).

We also obtained *in vitro* whole-cell intracellular recordings from ELp neurons in one clade-A species (*B*. *niger*) and from EL neurons in *P*. *tenuicauda*. We measured interval tuning in response to electrical microstimulation of the lateral lemniscus with pulse trains having IPIs from 10 to 100 ms. Out of seven neurons recorded in *B*. *niger*, five were characterized as high-pass, with strongest responses to short inter-pulse intervals, one as band-pass, and one as complex (Fig. 5c,d). In *P*. *tenuicauda*, six out of eight neurons were high-pass, one low-pass, and one band-pass (Fig. 5c,g). IPI-sensitive neurons of both species were morphologically similar, with extensive dendritic arbors (Fig. 5e,f,h,i).

### Hindbrain axonal projections to ELa follow a long and convoluted path, but hindbrain axonal projections to EL do not

**Figure 6:**
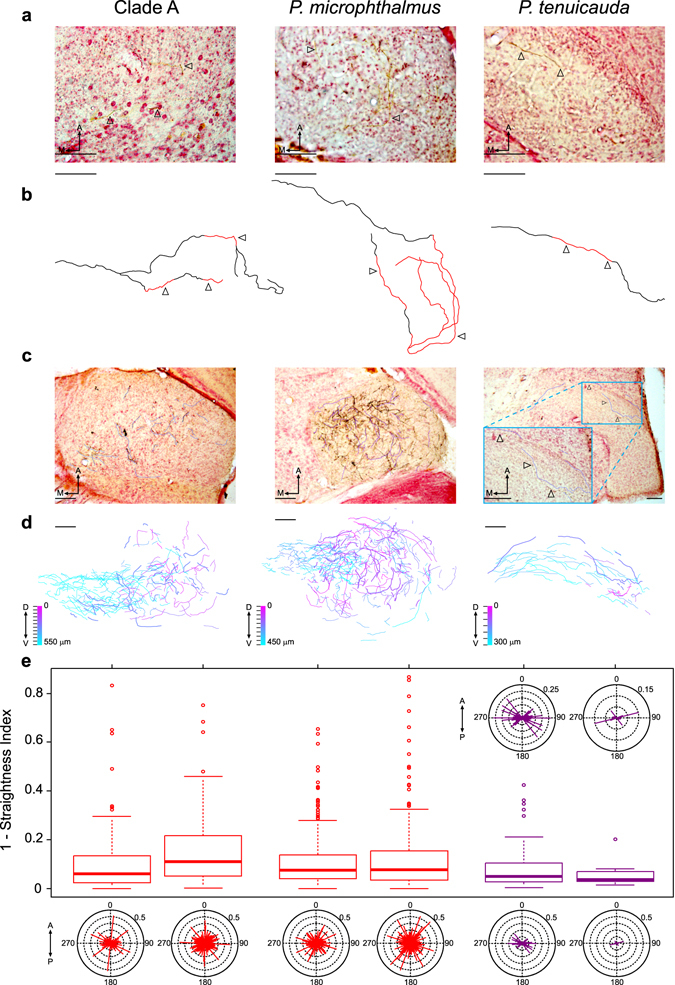
Axonal projections from hindbrain cells are long and follow a convoluted path after entering ELa, but not EL. (**a**) Representative photomicrographs of 50-µm horizontal sections of the brain at the level of ELa of a clade-A species (*B*. *brachyistius*, left) and of *P*. *microphthalmus* (center), and at the level of EL in *P*. *tenuicada* (right), depicting stained contralateral axonal projections (arrowheads). (**b**) Reconstruction of individual axons by tract tracing from 50-µm horizontal sections of the brain at the level of ELa of a clade-A species (left, *B*. *brachyistius*, 7 sections) and of *P*. *microphthalmus* (center, 4 sections), and at the level of EL of *P*. *tenuicauda* (right, 5 sections). Arrowheads in (**b**) indicate the stained axonal segments depicted in (**a**) and the red segments of the reconstructed axons in (**b**) were obtained from the corresponding section in (**a**), whereas the black segments were stained axonal segments from adjacent sections. (**c**) Representative photomicrographs and tracings of the axonal segments from one 50-µm horizontal section of the right ELa of a clade-A species (*B*. *niger*, left) and *P*. *microphthalmus* (center), and of the right EL of *P*. *tenuicauda* (right) used for calculating the angle of axonal projections and how much the axonal segments deviate from a straight line (calculated as 1- straightness index; see main text and Methods). Tracings in (**c**) are shifted to facilitate view of the traces and the associated labeled axonal segments. (**d**) Plots of axonal segments combined from all the photomicrographs in which labeled axonal segments were found, from the most dorsal section (magenta) to the most ventral section (cyan), in ELa of one clade-A species (*B*. *niger*, left) and *P*. *microphthalmus* (center), and in EL of *P*. *tenuicauda* (right). In (**d**), the axonal segments corresponding to the photomicrograph in (**c**) are plotted with a thicker line and the tick marks by the color scale represent the number of sections used to create the composite plot. (**e**) Box plots indicating how much axonal segments deviate from straight lines and circular plots of the angles of axonal segments in ELa (red) of two clade-A species (left) and two subjects of *P*. *microphthalmus* (center), and in EL (purple) of two subjects of *P*. *tenuicauda* (right). Box plots depict values of 1-straightness index; a value of zero represents a straight segment while values close to one represent highly convoluted segments. These same values are illustrated in the lengths of the lines in the circular plots, which are all centered on the origin at angles relative to the anterior-posterior axis. The two circular plots on top of the box plots for *P*. *tenuicauda* are close-up versions of the ones in the bottom of the figure. To generate the composite images in (**d**), photomicrographs with axonal segments were aligned by setting one reference line on an anterior-posterior axis of the brain through the medial division of the telencephalon, the optic tectum, the corpus cerebelli, and the eminentia granularis. A second reference line was set ortogonal to the anterior-posterior axis and placed anterior to the thickest part of the optic tectum. The photomicrograph in (**c**) and the composite plot of axonal segments in (**d**) for *P*. *microphthalmus* are from a subject in which a random sample of axonal segments were analyzed (see Methods). In (**e**), data for clade-A species come from one *B*. *brachyistius* (left, n = 118 axonal segments) and one *B*. *niger* (right, n = 236); sample sizes for *P*. *microphthalmus* are 310 (left) and 352 (right), and for *P*. *tenuicauda* are 68 (left) and 8 (right). Scale bars in all photomicrographs and tracings represent 100 µm. A: anterior. M: medial. D: dorsal. V: ventral. P: posterior.

Long and convoluted axonal projections of hindbrain axons within ELa establish delay lines that are critical for the mechanism by which small cells analyze EOD waveform in clade-A species^28, 29^. Here, we asked whether the axonal projections from hindbrain follow a long and convoluted path in all three lineages. We examined contralateral labeled axons after injections of neuronal tract tracers in the left ELa of clade-A species and *P*. *microphthalmus*, and in the anterior end of the left EL of *P*. *tenuicauda* (Figs 2 and 6). We tract-traced one axon per clade and found that the axons in clade A and *P*. *microphthalmus* have more branches, are longer, and follow a more convoluted path than the axon traced in *P*. *tenuicauda* (Fig. 6a,b; *B*. *brachyistius*: 1 axon with 3 branches of 1.11 mm, 0.77 mm, and 1.07 mm; *P*. *microphthalmus*: 1 axon with 3 branches of 0.99 mm, 1.01 mm, and 1.10 mm, *P*. *tenuicauda*: 1 axon without branches of 0.49 mm).

To investigate differences in axonal projections across lineages, we obtained three measurements from labeled axonal segments. We calculated a straightness index as the ratio of the Euclidian distance between the endpoints of the segment to the total length of the segment^38^. To compare how convoluted the axonal segments were across lineages, we subtracted this index from 1. A value of zero corresponds to a straight segment while values closer to one correspond to highly convoluted segments. We also calculated the angle between the line connecting the endpoints of the segment and the anterior-posterior axis of the brain (0° anterior, 180° posterior). We predicted that if axonal projections are targeted, then the angles of the axonal segments should be clustered around one general direction. Conversely, if axonal projections are convoluted, then the angles should be widely distributed. We also estimated a branching index as the number of nodes in which an axonal segment bifurcates, divided by the total number of axonal segments. The branching index is a relative measure in which a value of zero describes no axonal branching whereas values close to one correspond to high levels of branching.

We found differences across lineages in the straightness of the axonal projections (ANOVA: F_2,1089_ = 5.97, p = 0.0026; Fig. 6c–e), with straighter axonal segments in EL of *P*. *tenuicauda* (median value of 1-(straightness index) = 0.048) and more convoluted axonal segments in ELa of *P*. *microphthalmus* (0.075) and clade-A species (0.094) (Fisher’s LSD t-test pairwise comparisons: all p < 0.03). The angles of axonal segments also varied across lineages (Mardia-Watson-Wheeler: W = 26.32, p < 0.0001) and were restricted to a narrower range in EL of *P*. *tenuicauda* (Fig. 6c–e). Furthermore, axonal projections from the hindbrain show less branching in EL of *P*. *tenuicauda* than in ELa of clade-A species and *P*. *microphthalmus* (Axon branching indices: *B*. *brachyistius* = 0.034; *B*. *niger* = 0.051; *P*. *microphthalmus* subject 1 = 0.032; *P*. *microphthalmus* subject 2 = 0.07; *P*. *tenuicauda* subject 1 = 0.029; *P*. *tenuicauda* subject 2 = 0.0). Within lineages, these patterns were consistent regardless of whether axonal labeling was sparse or dense (Fig. 6). Together, these results suggest that incoming axonal projections follow a relatively long and convoluted path in ELa of clade-A species and *P*. *microphthalmus*, but not in EL of *P*. *tenuicauda*.

**Figure 7:**
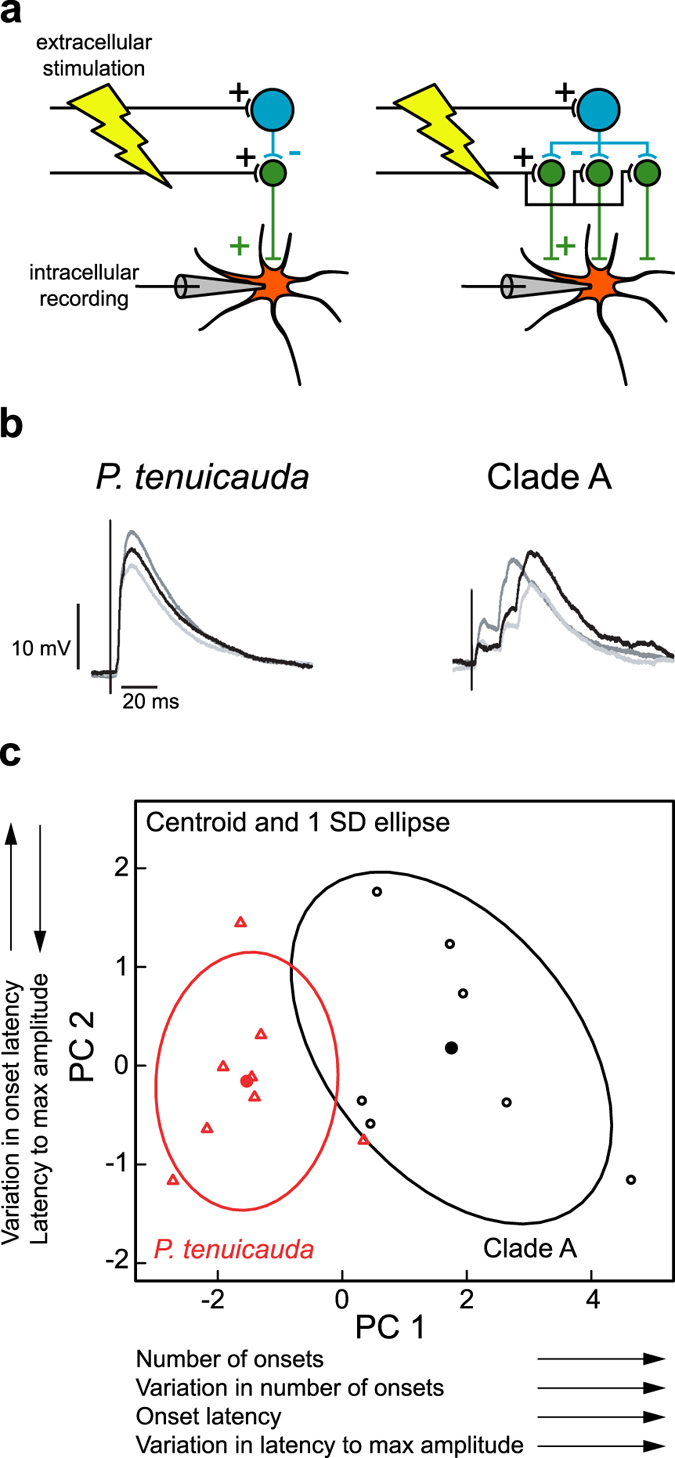
Multipolar cells in ELp receive excitatory inputs at more variable and longer latencies than multipolar cells in EL. (**a**) Schematic of the experiment designed to test whether incoming hindbrain axons act as delay lines in ELa, but not in EL. Extracellular stimulation of axons projecting from the hindbrain to the midbrain through the lateral lemniscus result in excitatory inputs to large inhibitory cells and to small cells. Small cells project to multipolar cells. The cartoon on the left depicts a simple excitatory-inhibitory circuit in which output from small cells to multipolar cells come shortly after stimulation of the lateral lemniscus. On the right, the circuit includes delay lines in the projections onto small cells. Because delay lines cause inputs to small cells to be delayed by different amounts, outputs from small cells to multipolar cells will be delayed by different amounts. Therefore, excitatory postsynaptic potentials (EPSPs) of multipolar cells should reveal several excitatory inputs at more variable latencies in circuits with delay lines. (**b**) Three traces of representative EPSPs recorded from one multipolar cell in EL of *P*. *tenuicauda* (left) and one multipolar cell in ELp of a clade-A species (*B*. *niger*, right). The EPSPs of the clade-A species do indeed reveal several excitatory inputs with a greater range of latencies than the EPSPs of *P*. *tenuicauda*. (**c**) The six variables used to describe the EPSP curves (see main text and Methods) can be summarized by two principal components. The first principal component (PC1) has an eigenvalue of 4.3, explains 72% of the variation, and loads heavily (>0.4) on the median and standard deviation in the number of onsets, the median latency of the onsets, and the standard deviation in the latency to the maximum amplitude of the EPSP. The second principal component (PC2) has an eigenvalue of 0.85, explains an additional 14% of the variation, and loads heavily (>0.45) on the median latency to reach the maximum amplitude of the EPSP and the standard deviation in the latencies of the onsets. Scores of the first principal component (PC1) were higher in *B*. *niger*, the clade-A species (black, n = 7), than in *P*. *tenuicauda* (red, n = 8). The current pulses used to obtain the traces in (**b**) were delivered at an amplitude of 160 µA for *P*. *tenuicada* and 120 µA for clade A (see Methods).

To directly test this hypothesis, we investigated whether hindbrain axonal projections to ELa, but not EL, establish functional delay lines. We obtained *in vitro* intracellular whole-cell recordings from multipolar cells, and measured responses to electrical microstimulation of the lateral lemniscus in one species with ELa/ELp (*B*. *niger*) and one species with undifferentiated EL (*P*. *tenuicauda*). If inputs to small cells follow delay lines in ELa but not EL, then this would result in a wider range of latencies and an overall greater delay in the synaptic inputs to multipolar cells in ELp (Fig. 7a).

Patterns of excitatory post-synaptic potentials (EPSPs) varied between species (Fig. 7b). In *P*. *tenuicauda*, EPSPs of multipolar cells were characterized by one or very few depolarization onsets shortly after the stimulus. In clade-A species, however, EPSPs were characterized by multiple onsets at longer and more variable latencies. Similarly, EPSPs reached the maximum amplitude at longer and more variable latencies in the clade-A species. To quantify these apparent differences, we ran a principal component analysis on six measurements from the EPSPs: the median and the standard deviation in the number of depolarization onsets, the median and the standard deviation in the latency of the onsets, and the median and the standard deviation in the latency to the maximum amplitude. Species differed in the first principal component (PC1; ANOVA: F_1,13_ = 26.31, p < 0.001; Fig. 7c), which loaded (>0.4) on the median and standard deviation in the number of onsets, the median latency of the onsets, and the standard deviation in the latency to the maximum amplitude of the EPSP. Thus, multipolar cells in ELp appear to receive several excitatory inputs at variable and longer latencies, while multipolar cells in EL receive only a few inputs at short latencies. The latencies of excitatory inputs to ELp multipolar cells are consistent with small-cell first-spike latencies in ELa in response to electrosensory stimulation, which are longer and more variable than first-spike latencies of ELa large cells and nELL cells^29, 34^. Together, these results support the hypothesis that axonal projections act as delay lines in ELa, but not in EL.

## Discussion

**Figure 8:**
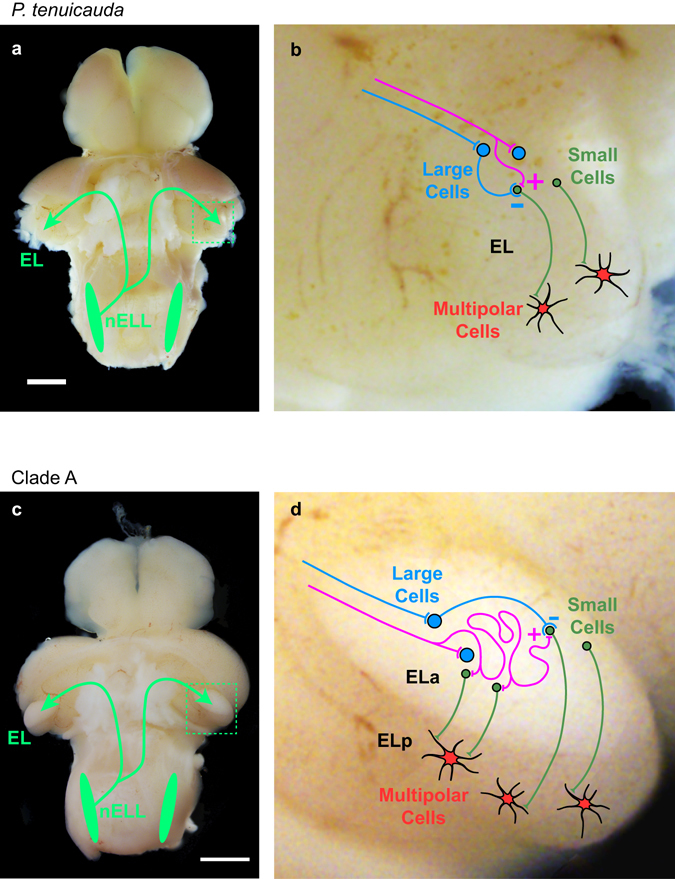
Schematic of the knollenorgan electrosensory pathway of species with small and undifferentiated EL and species with enlarged and subdivided ELa/ELp. (**a**) Photograph of the brain of *P*. *tenuicauda* (**a**) and *B*. *niger* (**c**) with the valvula cerebellum removed to expose the hindbrain and midbrain. Neurons in the electrosensory lateral line lobe (nELL) in the hindbrain project bilaterally to the exterolateral nucleus of the midbrain (EL). In clade-A species and *P*. *microphthalmus*, EL is enlarged and subdivided into anterior (ELa) and posterior regions (ELp). Enlarged photograph of EL (**b**) and ELa/ELp (**d**) with a schematic of the neural circuits. In species with a small and undifferentiated EL (**b**), axons of nELL cells synapse onto two types of cells in the lateral lemniscus and the anterior end of EL: GABAergic cells (large cells) and adendritic small cells. Large cells project onto small cells, establishing an excitatory-inhibitory circuit by which small cells in EL likely perform signal location analysis based on a subtraction mechanism. Small cells project to multipolar cells in the posterior end of EL that perform inter-pulse interval analysis. In species with ELa/ELp (**d**) nELL axons synapse onto GABAergic cells (large cells) upon entering ELa and then follow a long and convoluted path, synapsing on several adendritic small cells throughout their length. Inhibitory large cells project onto small cells, establishing a delay-line anti-coincidence detection mechanism by which small cells in ELa perform EOD waveform analysis. Small cells in ELa project to multipolar cells in ELp that perform inter-pulse interval analysis. Scale bars = 1 mm.

We show that the neural circuits devoted to processing communication signals in EL and in ELa/ELp of clade-A species and *P*. *microphthalmus* share the same substrates (Fig. 8). Hindbrain cells project ipsi-, contra-, and bi-laterally to the midbrain through the lateral lemniscus with a contralateral bias. Large, GABAergic inhibitory cells synapse onto adendritic small cells with large, calyx-like terminals. These small cells project to IPI-sensitive multipolar cells. The main anatomical difference between EL and ELa/ELp circuitry appears to be that axonal projections from the hindbrain follow a long and convoluted path after they enter ELa, but not EL (Fig. 8). Electrophysiology suggests that this anatomical difference establishes functional differences in the timing of synaptic input (Fig. 7). We discuss our results in relation to this anatomical difference in the neural circuit and its functional significance for sensory processing of communication signals.

The evolution of ELa/ELp, and the concomitant ability to detect EOD waveform variation, may be partly due to the lengthening of incoming axons from the hindbrain. Long and winding axonal projections from the hindbrain are essential for the temporally precise comparisons of peripheral spike times that allow for EOD waveform analysis in clade-A species^28, 29^. These convoluted axonal projections establish variation in the relative timing of excitatory and inhibitory input across the population of small cells and underlie the delay-line anti-coincidence detection mechanism by which submillisecond time differences in EOD waveform are processed^28, 29^. Our results suggest that long and convoluted axonal projections from the hindbrain to ELa are also present in the *P*. *microphthalmus* lineage, thus conferring a similar ability to detect subtle variation in EOD waveform.

Delay lines established by long axonal projections lead to a greater range of timing comparisons being processed in ELa, and this likely requires more cells in the circuit. We show that EL and ELa/ELp of clade-A species and *P*. *microphthalmus* contain the same three basic types of neurons: small cells, large inhibitory cells, and multipolar cells. Because ELa/ELp is more than twice the size of EL^12^, and the three cell types have similar sizes in all three lineages (Fig. 4), the enlarged ELa/ELp likely relates to greater numbers of these cells. We propose that the evolution of ELa/ELp and the ability to detect EOD waveform variation is associated with an elongation of axons to form delay lines and an increase in the number of cells needed to process the resulting timing information.

Differences between the microcircuitry in ELa and the anterior end of EL likely reflect differences in the type of sensory information processed in these brain regions. The perceptual ability to detect differences in EOD waveform depends on the capacity of the electrosensory system to process submillisecond time differences between different edges of the EODs. Knollenorgans on one side of the body respond to rising edges of an EOD, while knollenorgans on the opposite side of the body respond to falling edges^14, 16, 33^. This causes small cells in ELa to receive inhibition from large cells elicited by one edge of the EOD and delayed excitation by another edge of the EOD^22, 28, 29^. Variation in the length of excitatory axonal projections onto small cells establishes variation in selectivity to the duration between EOD edges, thereby recoding a temporal code into a population code.

In contrast, homogeneity in excitatory axonal projections in EL would establish homogeneity in small-cell temporal selectivity. This suggests that small cells in the EL circuit cannot perform EOD waveform analysis. Indeed, unlike the spiking receptors of species with an ELa/ELp, the oscillatory receptors of species with an EL do not encode EOD waveform into small timing differences^14^. Instead, small cells in EL may process excitation and inhibition to perform signal localization. The oscillating knollenorgans in these species respond to electrosensory stimuli by resetting their phase^14^. The phase resets of knollenorgans on opposite sides of the body are 180° out of phase with each other^14^. Thus, receptors on one side of the body reset to a peak first, and receptors on the opposite side reset to a trough first. This difference in reset phase encodes information about stimulus location. Further, if receptors on one side of the body trigger excitation and receptors on the opposite side of the body trigger inhibition, small cells in EL could determine signal location through a subtraction mechanism. In one hemisphere of the brain, stimuli coming from one direction would elicit maximal excitation from one side of the body and minimal inhibition from the opposite side of the body. Stimuli coming from the other side would elicit maximal inhibition and minimal excitation. Responses in the other brain hemisphere would presumably mirror this pattern. Thus, the ancestral excitatory-inhibitory circuit in EL may have originally evolved to serve signal localization, and the addition of axonal delay lines in clade A and *P*. *microphthalmus* appears to be an elaboration on this basic motif that established the novel ability to analyze signal waveform.

Considering our current understanding about the evolution of this group of fishes^12^, we show here that (i) the ancestral neural circuit devoted to processing communication signals contains all the building blocks necessary to detect signal variation, and (ii) similar changes in this neural circuit occurred in parallel in two lineages able to detect signal waveform variation. Thus, our results show that a relatively small change to a simple excitatory-inhibitory circuit (axonal elongation) can establish temporally precise computations that underlie the evolution of a novel sensory perceptual ability. To the best of our knowledge, this is the first study to elucidate the underlying physiological and anatomical substrates for evolutionary change in sensory perception at cellular and circuit levels.

## Methods

### Animals

Our protocols for housing, handling, and testing animals adhered to the guidelines established by the National Institutes of Health and were approved by the Institutional Animal Care and Use Committee at Washington University in St. Louis. For details about animal housing and handling, readers are referred to Baker *et al*.^14^.

### *In vivo* electrophysiology: evoked field potentials

Our protocols to obtain *in vivo* evoked field potentials have been previously described and readers are referred to those studies for details not included here^21, 30, 39^. Briefly, we first anesthetized the fish with 300 mg/ml of tricaine methanesulfonate (MS-222, Sigma-Aldrich, St. Louis, MO) and paralyzed it with 100–150 µl of a 3 mg/ml solution of gallamine triethiodide (Flaxedil, Sigma-Aldrich, St. Louis, MO). Then, we transferred the fish to a recording chamber (20 × 12.5 × 45 cm) filled with fresh water, leaving a small region of the left side of the head above the water level. During surgery, we maintained general anesthesia by respirating the fish with an aerated solution of 100 mg/ml MS-222 through a pipette tip in the mouth. We then applied 0.4% Lidocaine as a local anesthetic on the surgery site and removed the skin, secured a post to the skull, and removed part of the skull to expose ELa and ELp in clade-A species and *P*. *microphthalmus*. Because the smaller EL of *P*. *tenuicauda* is not exposed as in the other species, we exposed it by separating the medial edge of the optic tectum and lateral edge of the valvula cerebelli with two retractors made of borosilicate capillary glass. We brought the fish out of general anesthesia after surgery by switching to aerated freshwater respiration. While flaxedil silences the EOD, the EOD command from spinal electromotor neurons can be recorded. We monitored the fish’s electromotor output with a pair of electrodes next to the fish’s tail and the EOD commands from spinal electromotor neurons were amplified 1000 × (A-M systems, Model 1700) and sent to a window discriminator for time-stamping (SYS-121, World Precision Instruments).

After the fish had recovered from anesthesia, we recorded evoked field potentials with electrodes made of borosilicate capillary glass (o.d. = 1.0 mm, i.d. = 0.5 mm; A-M Systems, Model 626000). Electrodes were pulled on a Flaming/Brown micropipette puller (Sutter Instruments Company, Model P-97), broken to a tip diameter of 10–15 µm, and filled with 3 M NaCl. Evoked potentials were amplified 1000× and band-pass filtered between 0.01 and 5 kHz (A-M systems, Model 1700), digitized at a rate of 97.6 kHz (Tucker Davis, Model RX 8), and saved using custom software in Matlab (Mathworks, MA, USA). For each type of stimulus, we recorded responses to 10 repetitions. The midbrain EL in *P*. *tenuicauda* and ELa/ELp in clade-A species and *P*. *microphthalmus* are brain regions devoted to processing communication signals from other individuals; when an EOD command is generated, an inhibitory corollary discharge blocks electrosensory responses to the fish’s own EOD in the hindbrain nELL^18–21, 34^. Therefore, we ignored and repeated all repetitions in which a fish produced an EOD command two to five milliseconds before the stimulus.

Transverse electrosensory stimuli were delivered through three vertical electrodes on each side of the recording chamber. We generated digital stimuli in Matlab (Mathworks, MA, USA), converted to analog and delivered with a Model RX8 signal processor (Tucker-Davis), attenuated with a PA5 attenuator (Tucker-Davis), and isolated from ground with a stimulus isolation unit (A-M Systems, Model 2200).

To investigate whether the contralateral bias in hindbrain projections to the midbrain result in more sensory information reaching the midbrain from the contralateral side of the fish’s body, we measured evoked potentials in response to long square pulses for which the responses to each edge of the pulse can be separated. Square pulses had a duration of 50ms and were delivered with an amplitude of 34.6 mV/cm. The anodal stimulus electrodes were set to either the left side or the right side of the fish’s body for stimuli with normal and reversed polarity, respectively. When the anodal electrodes were set to the left, knollenorgans on the left side of the fish’s body (ipsilateral to recording electrode in the midbrain) were stimulated with the leading edge of the square pulse, while knollenorgans on the right side (contralateral) were stimulated with the trailing edge. When the anodal electrodes were set to the right, the right side of the body (contralateral) was stimulated with the leading edge of the square pulse and the left side of the body (ipsilateral) with the trailing edge. We measured the peak-to-peak amplitude of the mean evoked potential in response to each edge of the square pulse and normalized them to the maximum value. In clade-A species and *P*. *microphthalmus*, mean evoked potentials were normalized separately for ELa and ELp. We used repeated-measures ANOVA in R^40^ to investigate the effect of the side of the body stimulated with respect to the recording electrode (contralateral vs. ipsilateral) and the effect of order of stimulation (leading vs. trailing edge of the square pulse).

To examine inter-pulse interval (IPI) processing in the central electrosensory system, we asked whether evoked field potentials attenuated in response to pulse trains in ELa and ELp of clade-A species and *P*. *microphthalmus* and in EL of *P*. *tenuicauda*. We obtained evoked potentials in response to trains of 10 bipolar square pulses with inter-pulse intervals of either 10, 20, 30, 40, 50, 60, 70, 80, 90, or 100 ms. These values are within the range of natural variation in IPIs during social interactions^32^. Bipolar square pulses were 0.5-ms long, had an amplitude of 73.6 mV/cm, and were delivered with normal polarity (peak preceding trough) after setting the anodal stimulating electrodes to the left of the fish’s body. From the mean evoked potential obtained for each pulse train, we measured the peak-to-peak amplitude in response to each of the 10 pulses. For each pulse train, we measured attenuation relative to the evoked potential response to the first pulse in the pulse train. Thus, we measured the average peak-to-peak amplitude of the second to tenth pulse and then normalized it to the peak-to-peak amplitude of the evoked potential in response to the first pulse of the pulse train. In clade-A species and in *P*. *microphthalmus*, we used repeated-measures ANOVA to investigate the effects of inter-pulse interval and nucleus (ELa vs. ELp) on the normalized evoked potentials. In *P*. *tenuicauda*, we analyzed the effect of inter-pulse interval on the normalized amplitude of the evoked potentials and we added the latency to the evoked potential response to single stimulus pulses as a covariate in the model as a proxy of where in the anterior-posterior axis of EL the recordings were obtained.

### *In vitro* electrophysiology: whole-cell recordings

We used an *in vitro* whole-brain preparation recently developed in our lab, and readers are referred to those studies for additional details^36, 41^. Briefly, fish were anesthetized in 300 mg/L of MS-222 and then transferred to a container with ice-cold, oxygenated artificial cerebrospinal fluid (ACSF; composition in mM: 124 NaCl, 2.0 KCl, 1.25 KH_2_PO_4_, 24 NaHCO_3_, 2.6 CaCl_2_, 1.6 MgSO_4_ × 7H_2_O, and 20 glucose, pH = 7.45; osmolarity = 310 mOsm) that contained 1 mM kynurenic acid (KA) to reduce potential excitotoxicity. We performed a craniotomy and removed the valvula cerebelli by suction. We then removed the rest of the brain from the skull and allowed it to equilibrate in oxygenated ACSF containing 0.5 mM KA at room temperature for one hour. The brain was then transferred to a recording chamber (Model RC-26GPL,Warner Instruments, Hamden, CT, USA) and secured by two slice anchors (Model SHD-26GH,Warner Instruments) placed below (ventral) and above (dorsal) the brain. We then placed the chamber on a recording platform (Burleigh Gibraltar; EXFO, Mississauga, ON, Canada) where the brain was continuously perfused at room temperature with oxygenated ASCF at a rate of approximately 1 ml/min. The brain was perfused for one hour before starting recordings, to allow the KA to wash out.

We used a matrix array of stimulus electrodes to stimulate afferent excitatory inputs to EL of *P*. *tenuicauda* and ELa of one clade-A species. The matrix electrode array (Model MX42ABW, FHC, Bowdin, ME, USA) consisted of four channels of bipolar stimulation (eight electrodes total), and was placed in the lateral lemniscus located just medial and posterior to EL of *P*. *tenuicauda* and ELa of one clade-A species. We stimulated the lateral lemniscus with single pulses and with pulse trains of 10 pulses delivered with inter-pulse intervals between 10 and 100 ms, in 10 ms steps. Stimuli were bipolar square current pulses of 100 µs in duration and delivered at an amplitude of 50–200 µA using four separate isolated pulse generators (Model 2100; A-M systems, Sequim, WA, USA). To prevent overstimulation, we found the minimum number of stimulus channels and lowest stimulus amplitude that elicited strong and reliable responses for each recording. These stimulus settings were then used for all subsequent recordings.

We used transmitted light microscopy in an upright microscope (Model: BX51WI, Olympus, Tokyo, Japan) coupled to an EMCCD camera (Model: iXon Ultra 897, Andor, Belfast, Northern Ireland) to visualize neurons in ELp of clade-A species (*B*. *niger*: n = 2 fish, 7 neurons) and EL of *P*. *tenuicauda* (n = 3 fish, 8 neurons). We used filamented, borosilicate patch pipettes (o.d. = 1.00 mm, i.d. = 0.58 mm; A-M Systems, Model 601000) with tip resistances of 4–8 MΩ. The internal solution in the electrode consisted of (in mM) 130 K gluconate, 5 EGTA, 10 HEPES, 3 KCl, 2 MgCl_2_, 4 Na_2_ATP, 5 Na_2_ phosphocreatine, and 0.4 Na_2_GTP, and had a pH of 7.3–7.4 and an osmolarity of 280–290 mOsm. In addition, we added 0.1 mM of either Alexa Fluor 488 hydrazide (A10436, Life Technologies) or Alexa Fluor 568 hydrazide (A10437, Life Technologies) for fluorescent visualization. Whole-cell recordings were amplified (Model: MultiClamp 700B amplifier, Molecular Devices, Union City, CA, USA), digitized at a sampling rate of 60 kHz (Model Digidata 1440 A, Molecular Devices), and saved (Clampex v10.2, Molecular Devices). After recordings, we iontophoretically injected Alexa Fluor with hyperpolarizing current of −20pA for 2–10 minutes. We visualized neurons labeled with Alexa Fluor using a confocal imager (Model: X light, Crest optics, Roma, Italy) coupled to the EMCCD camera and a fluorescence illumination lamp (Model: Spectra X, Lumencor, Beaverton, OR, USA). We used Metamorph NX (Molecular Devices) and a motorized stage controller (Model: ProScan III, Prior Scientific, Rockland, MA, USA) to obtain z-stack photographs (in steps of 1 µm) of labeled neurons. Images of labeled neurons are based on maximum intensity projections of 120 to 212 photographs.

We used previously described protocols to construct inter-pulse interval tuning curves of single neurons^30, 35, 36, 41^. Briefly, we recorded five repetitions of responses to pulse trains consisting of 10 pulses with inter-pulse intervals between 10 and 100 ms (in 10-ms steps). The order in which pulse trains of particular IPIs were delivered was chosen at random. Traces in which synaptic stimulation elicited spikes were first filtered with a median filter of 1.5ms to remove spikes. We obtained the mean response across repetitions of each pulse-train stimulus. From the mean response to each IPI, we averaged the maximum depolarizations in response to the 2nd through 10th pulses. We then found the maximum value of these averages across all 10 IPIs and used it to normalize the averaged maximum depolarizations for all 10 IPIs. We used a threshold of 85% of the maximum response and linear interpolation to categorize the IPI tuning curve of each neuron. If the tuning curve crossed the 85% threshold in one point, the neuron was categorized as “low-pass” if long IPIs (i.e., IPIs greater than the IPI at threshold) elicited responses above 85%, or as “high-pass” if responses above 85% were elicited by short IPIs (i.e., IPIs lower than the IPI at threshold). If tuning curves crossed the 85% threshold in two points, neurons were classified as “band-pass” or “band-stop”, depending on whether responses in between both threshold crossings were above or below the 85% threshold, respectively. In this study, we did not find band-stop neurons. If the tuning curve crossed the 85% threshold more than twice, the neuron was classified as “complex”.

To investigate the timing of excitatory inputs onto multipolar cells in EL and ELp, we analyzed excitatory post-synaptic potentials (EPSPs) in response to single pulses. We obtained 10–20 repetitions for each neuron. From the response trace of each repetition, we measured (i) the latency to the maximum value of the EPSP, (ii) the number of onsets of depolarizations, and (iii) the latency to each depolarization onset. To find the depolarization onsets in response to the stimulus, we first filtered the trace obtained from each repetition with a median filter of 1.5 ms. We then calculated the first derivative of the filtered trace and filtered it with a moving average filter of 1.5 ms. Then, we obtained the second derivative of the trace and filtered it again with a moving average filter of 1.5 ms. To get an estimate of the baseline noise of the second derivative, we calculated the mean and standard deviation of the filtered second derivative during 5 ms before stimulus onset. We defined a depolarization onset as each occurrence in which the second derivative crossed, in an ascending manner, a threshold set to three standard deviations above the mean baseline noise. For each trace, we obtained (i) the maximum EPSP, (ii) the latency to the maximum EPSP, (iii) the number of depolarization onsets, and, with the latency to each onset, we calculated (iv) the median and standard deviation of onset latency. For each neuron, we calculated (i) the median number of onsets across traces, (ii) the standard deviation in the number of onsets across traces, (iii) the median latency to the maximum EPSP across traces, (iv) the standard deviation of the latency to maximum EPSP across traces, (v) the median across traces of the median depolarization onset latency of each trace, and (vi) the median across traces of the standard deviation of onset latency of each trace. We then ran a principal components analysis on these six variables obtained from each neuron and tested whether principal components differed between species using ANOVA.

### Neuronal morphology and projections

We used iontophoretic injections of neurobiotin or biocytin to investigate neuronal morphology and projections within the electrosensory pathway. Neuronal tract tracers were injected after recording evoked potentials *in vivo*. To inject the neuronal tract tracers, we used electrodes made of borosilicate capillary glass (o.d. = 1.0 mm, i.d. = 0.5 mm; A-M Systems, Model 626000) pulled on a micropipette puller (Sutter Instruments Company, Model P-97), broken to a tip diameter of 30–40 µm, and filled with a solution of 5% Neurobiotin or Biocytin in 0.01 M NaCl. We injected a DC current of 3–5 µA for 5–15 min (see below), using a Model A365 stimulus isolator (World Precision Instruments, Sarasota, FL, USA). Survival time after injections varied between 3 hours (for injections in ELp of clade-A species and *P*. *microphthalmus*) and 8 hours (for injections in EL of *P*. *tenuicauda* and injections in ELa of clade-A species and *P*. *microphthalmus*). After the survival period, fish were deeply anesthetized in MS-222 and perfused through the heart with ice-cold heparinized Hickman’s Ringer, followed by ice-cold fixative of 4% paraformaldehyde and 1% glutaraldehyde in 0.1 M phosphate buffer (PB). The brain was then removed from the skull, post-fixed overnight in the same fixative at 4 °C, and embedded in a gelatin block. We fixed the gelatin blocks in the same fixative overnight at 4 °C and cut them into 50 µm horizontal sections in ice-cold 0.1 M PB using a vibrating microtome (Ted Pella, Inc, D.S.K., model DTK-1000). Sections were then rinsed in 0.02 M PBS (2 × 10 min), followed by incubation in 0.05% hydrogen peroxide in 0.02 M PBS (1 × 10 min). We then rinsed the sections in 0.02 M PBS (3 × 10 min) and incubated them overnight at room temperature with an avidin-biotinylated horseradish peroxidase complex (ABC Elite Kit, Vector Laboratories) in 0.3% Triton in 0.02 M PBS. Sections were then rinsed again with 0.2 M PB (4 × 10 min) at room temperature. We then used a diaminobenzidine (DAB) reaction of 15 minutes in 0.5 mg/ml DAB in 0.1 M PB, followed by an additional 5–10 minutes after adding 0.002% H_2_O_2_ to visualize the label. Sections were then rinsed in 0.1 M PB (4 × 10 min) at room temperature, mounted on slides subbed with chrom-alum, counterstained with Neutral Red, dehydrated in an alcohol series of 50%, 70%, 90%, and 100% EtOH, cleared in three steps of xylenes, and coverslipped with Eukitt (Sigma). Using transmitted light microscopy in an upright microscope (Model: BX51WI, Olympus, Tokyo, Japan), we then analyzed the histological preparations as described below.

To investigate hindbrain projections to the midbrain, we injected neuronal tract tracers into the left ELa of clade-A species (*B*. *brachyistius*: n = 1, *B*. *niger*: n = 3, *G*. *petersii*: n = 1) and *P*. *microphthalmus* (n = 2) and into the anterior end of the left EL in *P*. *tenuicauda* (n = 3). After the iontophoretic injection of the neuronal tract tracer, we allowed 8 hours of survival time and proceeded to process the brain as described above. We then counted all labeled somas in the left and right nELL of the hindbrain and calculated the proportion of contralateral labeled cells. These brains were also used to search for bilateral axonal projections from the hindbrain to the midbrain by looking for labeled axons in the right ELa of clade-A species and *P*. *microphthalmus*, and in the right EL of *P*. *tenuicauda*. By tract-tracing, we reconstructed one contralateral axon per clade (*B*. *brachyistius*, *P*. *microphthalmus*, *P*. *tenuicauda*), from the first contact with a large cell to the end of all the axon branches, and we measured the length of each branch using the software DP2-BSW (Olympus). In addition, in all samples in which labeled axon segments were identified (n = 1 *B*. *brachyistius*, 1 *B*. *niger*, 2 *P*. *microphthalmus*, 2 *P*. *tenuicauda*), we tract-traced all the labeled segments using the software DP2-BSW (Olympus). For one *P*. *microphthalmus* in which over 1000 axon segments were labeled, we randomly chose 352 segments to measure; for the other 5 specimens, we measured all the labeled axon segments. We used the software DP2-BSW (Olympus) to measure the total length of each segment and the Euclidian distance between the endpoints of each segment. We first calculated the straightness index described by Batschelet (1981)^38^ as the length between endpoints of the segment divided by the total length of the segment. We then subtracted the straightness index from one; thus, a value of zero represents a straight line whereas values close to one represent highly convoluted segments. We compared how convoluted the axonal segments are across the three lineages (i.e., clade-A species, *P*. *microphthalmus*, and *P*. *tenuicauda*) using ANOVA in R^40^. We also estimated the direction of the axonal segments by measuring (DP2-BSW; Olympus) the angle between the line connecting the endpoints of the segment and the anterior-posterior axis of the brain, with 0° set to the anterior end of the brain and 180° to the posterior end. We used Mardia-Watson-Wheeler tests in Oriana v. 2.02 (Kovach Computing Services, Anglesey, Wales) to compare the distribution of segment direction angles across the three lineages. Finally, we searched for nodes in which the axonal segments bifurcate into different branches and calculated a branching index as the number of nodes divided by the total number of branches. When one stained segment bifurcated, we considered it as two branches, when it did not bifurcate, we considered it as one branch.

To investigate projections from ELa to ELp, and projections from the anterior to posterior end of EL, we injected neuronal tract tracers into the left ELp of three clade-A species (*B*. *brachyistius*: n = 1, *B*. *niger*: n = 1, *G*. *petersii*: n = 1) and of *P*. *microphthalmus* (n = 3) and in the posterior end of the left EL in *P*. *tenuicauda* (n = 3). We allowed a survival time of 3–4 hours before perfusion for clade-A species and *P*. *microphthalmus*, and of 8 hours for *P*. *tenuicauda*. In clade-A species and *P*. *microphthalmus*, we measured the diameter of all labeled cells in ELa. In *P*. *tenuicauda*, we measured the diameter of all adendritic labeled cells in EL and the lateral lemniscus. All measurements were obtained using transmitted light microscopy in an upright microscope (Model: BX51WI, Olympus) and DP2-BSW software (Olympus).

### GABA Immunohistochemistry

We used an antibody that was previously validated by Western blot and followed a protocol that has been successful in previous studies with mormyrids. Readers are referred to those studies for details not provided here^35, 42^. We obtained data from three individuals of clade A (n = 1 *B*. *brachyistyius*, n = 1 *B*. *niger*, n = 1 *P*. *adspersus*), three individuals of *P*. *microphthalmus*, and three individuals of *P*. *tenuicauda*. Fish were deeply anesthetized in MS-222 and then perfused through the heart with ice-cold heparinized Hickman’s Ringer, followed by ice-cold fixative of 4% paraformaldehyde and 0.3% glutaraldehyde in 0.1 M phosphate buffer (PB). After perfusion, the brain was removed from the skull, post-fixed overnight in the same fixative at 4 °C, and embedded in a gelatin block. Gelatin blocks were then fixed overnight at 4 °C in the same fixative and cut into 50-µm horizontal sections in ice-cold 0.1 M PB. We then incubated the sections for two hours at room temperature in a blocking solution of 4.5% normal goat serum, 0.3% Triton-X, and 0.3% bovine serum albumin in 0.1 M PB. Sections were then incubated at room temperature for 25 hours in a blocking solution containing (1:8000) a primary antibody against GABA coupled to bovine serum albumin with glutaraldehyde (Catalog No. 20094, Immunostar, Hudson, WI, USA). We then rinsed the sections in 0.02 M PB (4 × 10 min) and incubated them in blocking solution with goat anti-rabbit IgG biotinylated secondary antibody (1: 2000) at room temperature for four hours. Sections were then rinsed in 0.02 M PB (4 × 10 min) at room temperature, incubated with an avidin-biotinylated horseradish peroxidase complex (ABC Elite Kit, Vector Laboratories) at 4 °C overnight, and rinsed again with 0.2 M PB (5 × 10 min) at room temperature. To visualize the label, we then used a diaminobenzidine (DAB) reaction of 30 minutes in 0.5 mg/ml DAB in 0.1 M PB, followed by an additional 5–10 minutes after adding 0.002% H_2_O_2_. We then rinsed the sections in 0.02 M PB (4 × 10 min) at room temperature, mounted on slides subbed with chrom-alum, counterstained with Neutral Red, dehydrated in an alcohol series of 50%, 70%, 90%, and 100% EtOH, cleared in three steps of xylenes, and coverslipped with Eukitt (Sigma). For each brain, we used 3–5 sections as controls in which we did not add the primary antibody and therefore, yielded no cell-specific staining. We then used transmitted light microscopy in an upright microscope (Model: BX51WI, Olympus) and DP2-BSW software (Olympus) to measure the diameter of all stained cells in ELa and ELp of three clade-A species (*B*. *brachyistius*: n = 1, *B*. *niger*: n = 1, *P*. *adspersus*: n = 1) and *P*. *microphthalmus* (n = 3), and in EL and lateral lemniscus of *P*. *tenuicauda* (n = 3).

### Data availability

The datasets generated during the current study are available from the corresponding author on reasonable request.
